# The Relationship between Corvis ST Tonometry and Ocular Response Analyzer Measurements in Eyes with Glaucoma

**DOI:** 10.1371/journal.pone.0161742

**Published:** 2016-08-31

**Authors:** Masato Matsuura, Kazunori Hirasawa, Hiroshi Murata, Mieko Yanagisawa, Yoshitaka Nakao, Shunsuke Nakakura, Yoshiaki Kiuchi, Ryo Asaoka

**Affiliations:** 1 Department of Ophthalmology, University of Tokyo Graduate School of Medicine, Tokyo, Japan; 2 Orthoptics and Visual Science, Department of Rehabilitation, School of Allied Health Sciences, Kitasato University, Kanagawa, Japan; 3 Department of Ophthalmology, Saneikai Tsukazaki Hospital, Himeji, Japan; 4 Department of Ophthalmology and Visual Science, Hiroshima University, Hiroshima, Japan; Bascom Palmer Eye Institute, UNITED STATES

## Abstract

It is important to compare the results of Corneal Visualization Scheimpflug Technology instrument (CST) measurements and Reichert Ocular Response Analyzer (ORA) parameters. The purpose of the study was to investigate the association between CST measurements and ORA parameters in ninety-five patients with primary open-angle glaucoma. Measurements of CST, ORA, axial length (AL), average corneal curvature (CC), central corneal thickness (CCT) and intraocular pressure (IOP) with Goldmann applanation tonometry (GAT) were carried out. The association between CST and ORA parameters was assessed using linear regression analysis, with model selection based on the second order bias corrected Akaike Information Criterion index. Measurements from ORA (corneal hysteresis [CH] and corneal response factor [CRF]) had high intraclass correlation coefficients (ICC) and low coefficients of variation, but some CST parameters showed much lower reproducibility, namely: A1 length, A2 length, highest concavity time and peak distance. Of 12 CST parameters tested, 8 were significantly correlated with CH and 10 were significantly correlated with CRF, however, the magnitude of the correlation coefficients were weak to moderate at best. The optimal model to explain CH using CST measurements was given by: CH = -76.3 + 4.6*A1 time + 1.9*A2 time + 3.1 * highest concavity deformation amplitude + 0.016*CCT (R^2^ = 0.67, p <0.001). Similarly, the optimal model for CRF was given by: CRF = -53.5 + 4.2*A1 time + 1.9*A1 length + 20.8*A1 deformation amplitude + 0.8*A2 time + 0.017*CCT (R^2^ = 0.73, p <0.001). ORA parameters show higher reproducibility than CST measurements. Although many CST parameters are significantly related to ORA parameters, the strengths of these relationships are weak to moderate.

## Introduction

Glaucoma can severely damage a patient’s visual field and it remains the second leading cause of blindness worldwide, affecting approximately 60 million people.[1] The irreversible damage to visual function caused by this disease impacts on patients’ quality of life, so it is very important to detect glaucoma at an early stage and accurately predict its progression.

Intraocular pressure (IOP) is a well-established risk factor for the progression of glaucoma, [2–10] but other biomechanical measurements of the eye may also be useful determinants; in particular, research suggests that central corneal thickness (CCT) is correlated with glaucoma progression.[4,11] Furthermore, it has been shown that IOP measured with Goldmann applanation tonometry (GAT) is influenced by CCT.[12–24] A recent study, however, reported that low corneal hysteresis (CH), measured with the Ocular Response Analyzer (ORA, Reichert Ophthalmic Instruments, Depew, NY, USA), not CCT, is a risk factor of glaucoma.[25] This finding was later supported by a prospective research study[26] and a randomized controlled study.[27]

Recent advances in technology have enabled us to not only measure IOP, but also a number of other biomechanical properties of the eye. Such technologies include non-contact and impression applanation tonometry. The Ocular Response Analyzer is a non-contact tonometer that measures CH and also the corneal response factor (CRF); these two measurements represent different viscoelastic properties of cornea.[28] The Corneal Visualization Scheimpflug Technology tonometer (Corvis ST tonometry: CST; Oculus, Wetzlar, Germany) is an even newer instrument that allows quantitative and visual assessment of the biomechanical properties of cornea.[29] CST is also a non-contact tonometer, and is integrated with an ultra-high-speed Scheimpflug camera, enabling the direct visualization of corneal movement during the application of a rapid air-puff. Thus, both ORA and CST measure biomechanical properties of the cornea, however, the relationships between the different parameters derived from these two devices have not been reported. Therefore, the primary objective of this study is to investigate these relationships in a sample of patients with primary open angle glaucoma (POAG). A second purpose of the study is to explore the relationships between ORA and CST parameters against a number of other measurements, namely, axial length (AL), corneal curvature (CC), CCT, age and GAT-IOP.

## Method

The study was approved by Research Ethics Committee of the Graduated School of Medicine and Faculty of Medicine at The University of Tokyo. Written consent was given by patients for their information to be stored in the hospital database and used for research. This study was performed according to the tenets of the Declaration of Helsinki.

## Subjects

Ninety-five eyes of 95 POAG patients (53 males and 42 females) were included in this study. Inclusion criteria were: no abnormal eye-related findings except for POAG on biomicroscopy, gonioscopy and funduscopy. Eyes with the history of other ocular disease, such as age-related macular degeneration, and any intraocular surgery including cataract surgery were also excluded. Only subjects aged 20 years old or older were included and eyes with IOP>25 mmHg and contact lens wearers were excluded. Undiagnosed ocular hypertensive eyes were included. Subjects with diabetes mellitus were not included due to the possible effects of the disease on CH.[30–32] If both eyes satisfied the inclusion criteria, one eye was chosen at random to be the study eye.

## Corvis ST Tonometer Measurements

The principles of CST have been described in detail elsewhere;[29] CST parameters in the current study are inherited from those used in our previous report in which the relationship between CST Tonometry parameters and IOP, CCT and CC were investigated.[33] Briefly, the tonometer’s camera records a sequence of images of corneal deformation, capturing 4,330 images per second. CST measures CCT, deformation amplitude, applanation length and corneal velocity. Each measurement is further differentiated as follows: ‘A1/A2 time’ is the length of time from the initiation of the air puff to the first (cornea moves inwards) or second applanation (cornea moves outwards); ‘A1/2 length’ is the length of the flattened cornea at the first or second applanation; ‘A1/2 velocity’ is the velocity of the movement of cornea during the first or second applanation; ‘‘A1/2 deformation amplitude’ is the movement of the corneal apex of the flattened cornea at the first or second applanation; ‘peak distance’ is the distance between the two surrounding peaks of the cornea at the highest concavity; ‘highest concavity deformation amplitude' is the magnitude of movement of the corneal apex from before deformation to its highest concavity: ‘highest concavity time’ is the length of the time taken to reach highest concavity from pre-deformation of the cornea; ‘radius’ is the central curvature radius at the point of highest concavity.

The CST (software version; 1.2r1092) measurements were carried out three times on the same day, prior to the IOP-GAT measurement. Patients were given at least a one minute interval between each test. All CST measurements had sufficient reliability according to the “OK” quality index displayed on the CST monitor.

## Ocular Response Analyzer Tonometer Measurements

The Ocular Response Analyzer measures the central corneal response to indentation by a rapid jet of air, recording two applanation pressure measurements and two metrics of corneal biomechanics: CH and CRF.

The viscoelastic property of the cornea provides some resistance to the dynamic air puff of ORA, which causes a delay in the inward and outward applanation events; this delay captures the degree of resistance of the cornea and allows ORA to calculate the CH and CRF parameters.[34]

ORA measurements were carried out three times on the same day, prior to IOP-GAT measurement. The order of tests, ORA or CST, was decided randomly for each patient. All ORA data were of sufficient quality, as suggested by a quality index score of more than 7.5 for every test conducted.

## Other Measurements

GAT measurements were carried out after a topical anaesthesia of oxybuprocaine hydrochloride 0.4% (Benoxyl) with fluorescein staining. The tonometer was set at 10 mmHg before each reading. AL and CC were measured using the IOP master (Carl Zeiss Meditec). CCT was measured using CST.

## Statistical Analysis

The reproducibility of tonometer parameters was assessed using the coefficient of variation (CV) and the intraclass correlation (ICC) statistics. Correlation coefficients between the various CST parameters (IOP measured with CST, CCT, deformation amplitude, A 1/2 time, A 1/2 length, A 1/2 velocity, A 1/2 deformation amplitude, highest concavity time, and peak distance) and the five ocular/systemic parameters (GAT, CCT, AL, CC, and age) were calculated. The same correlation coefficients were also calculated for ORA parameters (IOP-CC, IOP-G, CH, and CRF). Finally, correlation coefficients between CST parameters and ORA parameters were determined.

Next, linear modelling was carried out to determine the optimal model to predict CH or CRF using CST measurements and the ocular/systemic parameters. The optimal model was selected from all possible combinations of predictors, a total of 2^17^ combinations (CH/CRF against age, GAT, AL, CCT, average CC, and the CST parameters of A 1/2 time, A 1/2 length, A 1/2 velocity, A 1/2 deformation amplitude, highest concavity time, highest concavity deformation amplitude, peak distance, and radius), based on the second order bias corrected Akaike Information Criterion (AICc) index, similarly to our previous report.[33] The AICc is a corrected version of the AIC, a common statistical measure used in model selection, which gives an accurate estimation even when the sample size is small.[35] As the degrees of freedom in a multivariate regression model decreases with a large number of variables, it is recommended to use model selection methods to improve the model fit by removing redundant variables.[36,37] All statistical analyses were performed using the statistical programming language ‘R’ (R version 3.2.3;The foundation for Statistical Computing, Vienna, Austria)

## Results

Characteristics of the study subjects are summarized in **Table 1**. The mean ± standard deviation (SD) [range] age of patients was 63.7±10.1 [41 to 86]. Fifty-three patients were male and 42 patients were female. Average GAT-IOP was 12.9±2.7 [8 to 22] mmHg and mean CCT was 531.3±34.6 [458.3 to 624.3] μm.

**Table 1:** Subject demographics.

variables	values
age, (mean ± sd) [range], years old	63.7 ± 10.1 [41–86]
male / female	53 (55.8%) / 42 (44.2%)
right / left	74 (77.9%) / 21 (22.1%)
GAT IOP, (mean ± sd) [range], mmHg	12.9 ± 2.7 [8–22]
AL, (mean ± sd) [range], mm	25.2 ± 1.6 [22.3–29.2]
average corneal curvature, (mean ± sd) [range], ms	7.7 ± 0.25 [7.2–8.2]
CCT, (mean ± sd) [range], μm	531.3 ± 34.6 [458.3–624.3]

sd: standard deviation, GAT IOP: intraocular pressure measured with Goldmann tonometry, AL: axial length, CCT: central corneal thickness

The reproducibility (ICC and CV values) of the CST and ORA parameters is summarized in **Table 2**. All ORA parameters obtained very high ICC values (0.80 to 0.91) and very low CV values (5.1 to 6.7). Many CST parameters demonstrated similar levels of ICC and CV, but some parameters were not so reproducible, including: A1 length (ICC = 0.44, CV = 2.7 ± 3.0), A2 length (ICC = 0.35, CV = 15.1 ± 10.0), highest concavity time (ICC = 0.36, CV = 3.0 ± 2.0), and Peak distance (ICC = 0.18, CV = 23.7 ± 19.0).

**Table 2:** Mean ± standard deviation of CST and ORA parameters and their intraclass correlation coefficient and coefficient of variation.

	Measurement				CV (%)	ICC
	1st	2nd	3rd	average		
IOP-corvis, mmHg	13.0 ± 2.4	12.8 ± 2.3	12.6 ± 2.5	12.7 ± 2.1	5.5 ± 3.0	0.86
	(6.5 to 20.5)	(6.5 to 25.0)	(6.0 to 26.0)	(8.7 to 22.0)	(0.0 to 15.7)	[CI: 0.82 to 0.91]
CCT-corvis, μm	531.9 ± 35.2	532.0 ± 34.9	532.3 ± 34.7	531.2 ± 34.4	0.7 ± 0.0	0.98
	(456.0 to 624.0)	(458.0 to 627.0)	(461.0 to 624.0)	(458.3 to 624.3)	(0.1 to 2.3)	[0.98 to 0.99]
A1 time, ms	7.2 ± 0.3	7.2 ± 0.3	7.1 ± 0.3	7.2 ± 0.3	1.1 ± 3.0	0.89
	(6.6 to 8.1)	(6.5 to 8.7)	(6.5 to 8.7)	(6.7 to 8.3)	(0.0 to 4.1)	[0.85 to 0.92]
A1 length, mm	1.72 ± 0.1	1.7 ± 0.1	1.7 ± 0.01	1.7 ± 0.1	2.7 ± 3.0	0.44
	(1.3 to 1.9)	(1.3 to 1.9)	(1.3 to 1.9)	(1.4 to 1.8)	(0.1 to 18.2)	[0.32 to 0.56]
A1 velocity, m/s	0.2 ± 0.002	0.2 ± 0.01	0.2 ± 0.01	0.2 ± 0.01	4.9 ± 3.0	0.62
	(0.1 to 0.2)	(0.1 to 0.2)	(1.3 to 1.9)	(0.1 to 0.2)	(0.6 to 14.8)	[0.52 to 0.72]
A2 time, ms	21.8 ± 0.5	21.9 ± 0.5	21.9 ± 0.5	21.9 ± 0.4	0.8 ± 1.0	0.71
	(20.7 to 23.6)	(19.5 to 23.2)	(20.2 to 23.2)	(20.9 to 23.2)	(0.1 to 6.5)	[0.62 to 0.78]
A1 deformation amplitude, mm	0.1 ± 0.01	0.1 ± 0.01	0.1 ± 0.01	0.1 ± 0.01	3.6 ± 2.0	0.63
	(0.1 to 0.2)	(5.5 to 10.9)	(0.1 to 0.2)	(0.11 to 0.14)	(0.6 to 19.5)	[0.53 to 0.72]
A2 length, mm	1.7 ± 0.3	1.7 ± 0.3	1.6 ± 0.3	1.7 ± 0.2	15.1 ± 10.0	0.35
	(0.9 to 2.2)	(13.5 to 23.2)	(0.7 to 2.2)	(0.8 to 2.2)	(0.3 to 44.2)	[0.23 to 0.48]
A2 velocity, m/s	-0.39 ± 0.1	-0.4 ± 0.1	-0.4 ± 0.01	-0.4 ± 0.01	-11.0 ± 8.0	0.66
	(-0.2 to -0.6)	(-0.01 to -0.7)	(-0.1 to -0.7)	(-0.6 to -0.2)	(-56.7 to -0.5)	[0.57 to 0.75]
A2 deformation amplitude, mm	0.4 ± 0.1	0.4 ± 0.1	0.4 ± 0.1	0.4 ± 0.1	7.9 ± 7.0	0.68
	(0.2 to 0.7)	(0.2 to 0.7)	(0.2 to 0.7)	(0.3 to 0.6)	(0.2 to 50.6)	[0.59 to 0.77]
Peak Distance, mm	3.5 ± 1.3	3.3 ± 1.2	3.3 ± 1.2	3.2 ± 0.8	23.7 ± 19.0	0.18
	(2.0 to 5.8)	(2.0 to 5.6)	(1.7 to 5.7	(2.2 to 5.1)	(0.4 to 49.2)	[0.06 to 0.32]
Highest concavity time, ms	16.9 ± 0.7	16.9 ± 0.8	16.9 ± 0.8	17.0 ± 0.6	3.0 ± 2.0	0.36
	(14.1 to 18.7)	(14.8 to 18.5)	(14.3 to 18.5)	(15.4 to 18.4)	(0.0 to 11.8)	[0.23 to 0.49]
Highest concavity deformation amplitude, mm	1.1 ± 0.1	1.1 ± 0.1	1.1 ± 0.1	1.1 ± 0.1	3.3 ± 2.0	0.86
	(0.1 to 1.4)	(0.9 to 1.4)	(0.7 to 1.4)	(1.4 to 0.8)	(0.3 to 12.5)	[0.81 to 0.90]
Radius, mm	7.5 ± 1.0	7.5 ± 1.2	7.4 ± 1.0	7.4 ± 0.8	6.7 ± 4.0	0.65
	(2.0 to 5.8)	(2.0 to 5.6)	(6.7 to 11.0)	(5.9 to 10.3)	(0.6 to 19.5)	[0.55 to 0.74]
IOP CC, mmHg	14.7 ± 2.7	14.7 ± 3.0	14.6 ± 3.0	14.5 ± 1.5	6.7 ± 4.0	0.84
	(9.0 to 23.2)	(8.1 to 22.2)	(7.2 to 22.5)	(8.3 to 21.3)	(0.4 to 22.3)	[0.78 to 0.88]
IOP-G, mmHg	12.6 ± 3.0	12.5 ± 3.2	14.6 ± 3.0	12.4 ± 3.3	6.5 ± 4.0	0.91
	(5.5 to 21.0)	(5.2 to 20.9)	(7.2 to 22.5)	(3.8 to 20.1)	(0.7 to 21.4)	[0.88 to 0.94]
CRF	8.6 ± 1.5	8.5 ± 1.6	8.5 ± 1.5	8.5 ± 1.5	5.1 ± 4.0	0.90
	(5.3 to 13.0)	(4.3 to 14.1)	(4.8 to 13.2)	(5.3 to 13.0)	(0.5 to 25.0)	[0.86 to 0.93]
CH	9.3 ± 16.9	9.2 ± 1.4	9.3 ± 1.3	9.3 ± 1.2	5.3 ± 4.0	0.80
	(6.7 to 13.6)	(5.9 to 14.1)	(5.3 to 14.2)	(7.2 to 13.8)	(0.4 to 23.3)	[0.74 to 0.86]

All values were represented as mean ± standard deviation and (range) in upper and lower cell, respectively, except for ICC. ICC was represented as mean and [95% confidence interval] in upper and lower cell, respectively.

CST: Corvis ST tonometry, ORA: ocular response analyzer, CV: coefficient of variance, ICC: intraclass correlation, IOP: intraocular pressure, sd: standard deviation, CCT: central corneal thickness, CH: corneal hysteresis, CRF: corneal resistant factor

The correlations between CST and ORA measurements against GAT-IOP, CCT, AL, CC, and age are shown in **Table 3**. CRF was significantly related to GAT-IOP (R = 0.50, p <0.01), however, CH was not significantly correlated (R = 0.13, p = 0.22). CCT was significantly correlated to A1 time, A1 length, A1 velocity, A1 deformation amplitude, A2 length, A2 velocity, A2 deformation amplitude, highest concavity deformation amplitude, and radius. AL had a significant relationship with A2 velocity, A2 deformation amplitude, highest concavity time, and radius. Age had a significant relationship with A1 velocity and A2 deformation amplitude.

**Table 3:** Correlation coefficients (with significance levels) between CST/ORA parameters and ocular and systemic parameters.

	GAT (mmHg)	CCT (mm)	AL (mm)	CC (mm)	Age (years)
IOP-corvis (mmHg)	0.76**	0.33**	-0.28	-0.096	-0.060
CCT (mm)	0.19	-	-0.080	0.016	-0.028
A1 time (ms)	0.75**	0.33**	-0.031	-0.082	-0.063
A1 length (mm)	0.10	0.33**	0.12	0.25*	0.092
A1 velocity (m/s)	-0.55**	-0.21*	0.14	-0.16	-0.29**
A1 deformation amplitude (mm)	-0.72**	-0.29**	0.16	0.0064	0.027
A2 time (ms)	-0.72**	-0.13	-0.057	0.050	-0.032
A2 length (mm)	0.25*	0.53**	-0.11	0.014	0.080
A2 velocity (m/s)	0.53**	0.34**	-0.42**	-0.0087	0.14
A2 deformation amplitude (mm)	0.09	0.24*	-0.42**	-0.069	0.37**
Peak distance (mm)	-0.11	-0.18	-0.18	-0.12	0.10
Highest concavity time (ms)	-0.16	0.10	-0.31**	-0.085	0.15
Highest concavity deformation amplitude (mm)	-0.72**	-0.29**	0.16	0.0064	0.027
radius (mm)	0.34**	0.30**	-0.25*	0.19	0.20
IOP CC (mmHg)	0.60**	0.10	0.029	-0.062	0.030
IOP-G (mmHG)	0.68**	0.41**	0.0050	-0.038	0.016
CRF (mmHg)	0.50**	0.67**	-0.31	0.011	-0.0096
CH (mmHg)	0.13	0.62**	-0.049	0.050	-0.028

** denotes significant at the p <0.01 level and

* represents significant at the p <0.05 level.

CST: Corvis ST tonometry, ORA: ocular response analyzer

The relationships between CST parameters and CH/CRF are summarized in **Table 4**. Significant relationships were observed between CH and A1 time, A1 length, A2 length, A2 velocity, A2 deformation amplitude, peak distance, highest concavity time, and radius. Similarly, CRF was significantly correlated with CST parameters: A1 time, A1 length, A1 velocity, A1 deformation amplitude, A2 time, A2 length, A2 velocity, Peak distance, highest concavity amplitude and radius.

**Table 4:** Correlation coefficients (with significance levels) between CST parameters and ORA parameters.

	0043		CRF	
	coefficient	p value	coefficient	p value
A1 time (ms)	0.38**	<0.001	0.72**	<0.001
A1 length (mm)	0.28**	0.005	0.33**	0.001
A1 velocity (m/s)	-0.19	0.062	-0.39**	<0.001
A1 deformation amplitude (mm)	-0.16	0.130	-0.55**	<0.001
A2 time (ms)	0.083	0.430	-0.38**	<0.001
A2 length (mm)	0.48**	< 0.001	0.54**	< 0.001
A2 velocity (m/s)	0.35**	< 0.001	0.53**	<0.001
A2 deformation amplitude (mm)	0.24*	0.019	0.20	0.053
Peak distance (mm)	-0.2*	0.047	-0.29*	0.043
Highest concavity time (ms)	0.26*	0.011	0.032	0.760
Highest concavity deformation amplitude (mm)	-0.16	0.130	-0.55**	<0.001
radius (mm)	0.35**	< 0.001	0.43**	<0.001

** denotes significant at the p <0.01 level and

* represents significant at the p <0.05 level.

CST: Corvis ST tonometry, ORA: ocular response analyzer, IOP: intraocular pressure, sd: standard deviation, CCT: central corneal thickness, CH: corneal hysteresis, CRF: corneal resistant factor

The optimal model to describe CH was given by: CH = -76.3 + 4.6*A1 time (p <0.001) + 1.9*A2 time (p <0.001) + 3.1 * highest concavity deformation amplitude (p = 0.014) + 0.016*CCT (p <0.001): R^2^ = 0.67, p <0.001. The optimal model to describe CRF was: CRF = -53.5 + 4.2*A1time (p <0.001) + 1.9*A1 length (p = 0.10) + 20.8*A1 deformation amplitude (p = 0.13) + 0.8*A2 time (p = 0.0046) + 0.017*CCT (p <0.001): (R^2^ = 0.73, p <0.001) (see **Table 5**).

**Table 5:** Parameter coefficients in the optimal model to explain CH or CRF.

	CH		CRF	
	R	p value	R	p value
A1 time (ms)	4.6	<0.001	4.2	<0.001
A1 length (mm)			1.9	0.10
A1 deformation amplitude (mm)			20.8	0.13
A2 time (ms)	1.9	<0.001	0.80	0.005
highest concavity deformation amplitude (mm)	3.1	-0.014		
CCT (μm)	0.016	<0.001	0.017	<0.001
R^2^	0.67	<0.001	0.73	<0.001

The optimal model was selected from the variables of age, gender, GAT-IOP, AL, CCT, average corneal curvature, and the CST parameters of deformation amplitude, A 1/2 time, A 1/2 length, A 1/2 velocity, highest concavity time, peak distance and A 1/2 highest concavity deformation amplitude. The second order bias corrected Akaike Information Criterion index was used to select the optimal model. CCT: central corneal thickness, CH: corneal hysteresis, CRF: corneal resistant factor

## Discussion

In the current study, CST and ORA measurements were repeatedly carried out in 95 eyes of 95 patients with POAG. ORA parameters showed high reproducibility but the reproducibility of CST measurements varied greatly according to the parameter studied. Several CST parameters were significantly correlated with GAT-IOP, namely: A1/2 time, A2 length, A1/2 velocity, A1 deformation amplitude, highest concavity deformation amplitude and radius. The ORA-derived CRF measurement was also significantly correlated to GAT-IOP. Similarly, the following CST-derived parameters were significantly correlated with CCT: A1 time, A1/2 length, A1/2 velocity, A1/2 deformation amplitude, highest concavity deformation amplitude and radius. Both ORA-derived CH and CRF were also significantly correlated to CCT. Finally, as expected, many CST parameters were significantly correlated with CH and CRF measurements.

It has been reported that ORA parameters are reproducible, with ICC values ranging between 0.78 and 0.93.[38] Our study results strongly support this assertion, as indicated by **Table 2**. We previously reported the reproducibility of CST parameters in normative subjects, showing high reproducibility for a number of parameters, including CCT, IOP-C, A1 time, A2 time and maximum deformation amplitude.[33] These measurements also demonstrated good reproducibility in the current study. Indeed, in the current study, reproducibility was even higher than in our previous research. The reason for this is not clear, but may be attributed to a difference in the responsiveness of the subjects studied. The current study population consisted of POAG patients who have experienced IOP measurements many times and, consequently, they may be less nervous during ORA and CST examinations compared to normative subjects without experience of IOP measurements. A number of new CST parameters were presently studied: A1 deformation amplitude, A2 deformation amplitude and highest concavity deformation amplitude were not implemented in earlier versions of CST (current version: 1.2r1092), but all these parameters had high ICC values and low CV values.

A number of CST parameters measure the movement of the cornea in an axial direction (cornea to post pole), namely: A1/2 time, A1/2 velocity, A1/2 deformation amplitude and highest concavity time, while other CST parameters measure the movement of the cornea in the direction vertical to an axial direction (parallel to corneal surface): A1/2 length and peak distance. We previously observed that axial direction parameters tend to have better reproducibility than parameters parallel to the corneal surface [33] and this was also the case in the current study. We hypothesize that this is a direct result of the mechanism by which CST calculates it parameters; the instrument’s camera shoots 4,330 images per second at an angle of direction that is perfectly designed to detect corneal movement in the axial direction, but less suitable to monitor corneal movement in the direction parallel to the corneal surface.

In our previous report, we found that A1 time and A1 velocity were correlated to GAT-IOP.[33] A1 time and A1 velocity were also correlated to GAT-IOP in the current study, but a number of additional CST parameters were also found to correlate with GAT-IOP: A2 time, A2 length, A1/2 velocity and A1 deformation amplitude. Similarly, a larger number of CST parameters were correlated with CCT in the current study compared to our previous research results.[33] A possible reason for this finding is the wider range of GAT-IOP and CCT measurements observed in our POAG patients compared with our normative subjects; this may allow significant correlations to be detected in a smaller sample size. Further, as already stated, the greater test-experience of our POAG group may lead to more precise measurements (lower variability), which, again, may allow significant correlations to be detected in a smaller sample of patients. In the current study, among the two ORA parameters, CRF was significantly related to GAT-IOP, agreeing with previous reports.[13,28,38–41] On the other hand, CH was not correlated with GAT, which also agrees with previous research [13,39–41], but controversial.[38–40] In ORA, the magnitude of the applied air-puff is adjusted to minimize the effect of a person’s IOP on the magnitude of ORA’s corneal-related measurements. No similar adjustment is made in CST and this may explain why many CST parameters were found to be correlated to GAT.

CH and CRF measurements are known to decrease with increasing age.[13,42,43] In the current study, age was not significantly correlated with CH or CRF, however, the age range of our study population was narrower than in previous research (mean ± SD = 63.7 ± 10.1 years old, compared to 57.7 ± 15.1[13], 46.5 ± 21.0[42], and 46.7 ± 19.4[43] years old). This may also explain why fewer CST parameters were related to age (mean±SD = 63.7±10.1 years) in the present study, compared to our previous report (52.1±23.4 years).[33]

A number of previous studies have investigated the viscoelastic property of the cornea which is a measure of the energy absorbed during the ‘loading/unloading’ or stress/strain cycle of viscoelastic materials, represented by the ‘corneal hysteresis’ measurement in ORA; [13,34,38,44–51] however, we previously reported that CST-derived measurements of the cornea may also be associated with hysteresis of cornea (not necessarily measured with ORA). The current results add weight to this argument, finding significant relationships between ORA-derived CH and CRF measurements and many CST parameters. The optimal models to explain CH and CRF both included CCT and CST parameters, while GAT-IOP was not selected (see **Table 5**). It should be noted that it is not entirely appropriate to consider the relationship between CH/CRF and CST parameters by simply interpreting the correlation coefficients in **Table 4**; this is because CST parameters are closely inter-correlated and also CST parameters and ORA parameters are correlated with IOP. Considering the optimal models for CH and CRF, it is not surprising that CH and CRF are large when A1 time is large (slow); the mechanism to calculate the A1 time measurement is identical to that in ORA noncontact tonometry (the time to applanation is measured following an air-puff injection[52]). It is also not surprising that CH is positively associated with A2 time and highest concavity deformation amplitude, because a large amount of energy would be absorbed in these eyes. Similarly, CRF is positively associated with A2 time. It is of interest to consider that corneas with large CRF measurements are associated with large and deep applanation areas at the first applanation event and the second applanation occurs slowly, as indicated by the positive coefficients for A1 length, A1 deformation amplitude, and A2 time. A1 length and A1 deformation amplitude were included in the optimal model for CRF, however the p values for these CST parameters were larger than 0.05 (0.10 and 0.13). We didn’t exclude these parameters because the optimal model was selected using model selection with AICc (basing on log-likelihood), not using the significance of the parameters. As a result, these parameters were significant, but the importance may not be large, as suggested by the relatively large p values. These parameters may have smaller p values with larger sample population. A further study should be carried out to investigate this aspect. However, in general, correlations were moderate or weak (**Table 4**), so we speculate that CST parameters may reflect other aspects of corneal biomechanics that are not captured by CH and CRF measurements. This seems likely given the difference in the mechanisms of the measurements; ORA-derived CH and CRF are measured by analyzing the difference of air puff values at the inward and outward events, while CST measures the actual movement of the cornea during the inward and outward events. Further, recent studies have suggested that ORA-derived CH may not represent the ‘hysteresis of the cornea’.[53,54]

It is interesting to note that all CST parameters, except A2 deformation amplitude and highest concavity time, were significantly correlated with CRF, whereas only 8 out of the total 12 CST parameters were correlated with CH. In addition, CST parameters were more strongly correlated with CRF than they were with CH.

A limitation of the current study is the effect of anti-glaucoma eye drops on corneal biomechanical properties. It has been reported that anti-IOP agents can change the cornea’s biomechanical properties.[55–59] As patients were recruited from a real world glaucoma clinic, a non-negligible effect of eye drops could exist in the current study. We also did not measure and analyze the effect of trabeculectomy on CST and ORA measurements, which is also of real word clinical interest. Finally, a future study should be performed to investigate the usefulness of ORA and CST parameters as risk factors in the progression of glaucoma, because a recent study[60] has shown the significant relationship between CST measured highest concavity deformation amplitude and β-zone parapapillary atrophy which has been known to be a risk factor for glaucoma.[61–63] In this token, the reproducibility of CST parameters is important when assessing the risk of glaucoma at the clinical settings.

In conclusion, ORA parameters demonstrate good reproducibility, but some CST parameters are less reproducible. CST parameters are significantly related to ORA measurements, however, the strength of these relationships is relatively weak.

## Supporting Information

S1 File Data analyzed. (CSV)
